# The Regulation Effect of *α*7nAChRs and M1AChRs on Inflammation and Immunity in Sepsis

**DOI:** 10.1155/2021/9059601

**Published:** 2021-11-03

**Authors:** Song Hu, Yundong Wang, Hongbing Li

**Affiliations:** ^1^General Surgery Department of Ezhou Central Hospital of Hubei Province, 436000, China; ^2^Infectious Diseases Department of Ezhou Central Hospital of Hubei Province, 436000, China; ^3^Emergency Department of the First People's Hospital of Guiyang of Guizhou Province, 550002, China

## Abstract

The inflammatory storm in the early stage and immunosuppression in the late stage are responsible for the high mortality rates and multiple organ dysfunction in sepsis. In recent years, studies have found that the body's cholinergic system can spontaneously and dynamically regulate inflammation and immunity in sepsis according to the needs of the body. Firstly, the vagus nerve senses and regulates local or systemic inflammation by means of the Cholinergic Anti-inflammatory Pathway (CAP) and activation of *α*7-nicotinic acetylcholine receptors (*α*7nAChRs); thus, *α*7nAChRs play important roles for the central nervous system (CNS) to modulate peripheral inflammation; secondly, the activation of muscarinic acetylcholine receptors 1 (M1AChRs) in the forebrain can affect the neurons of the Medullary Visceral Zone (MVZ), the core of CAP, to regulate systemic inflammation and immunity. Based on the critical role of these two cholinergic receptor systems in sepsis, it is necessary to collect and analyze the related findings in recent years to provide ideas for further research studies and clinical applications. By consulting the related literature, we draw some conclusions: MVZ is the primary center for the nervous system to regulate inflammation and immunity. It coordinates not only the sympathetic system and vagus system but also the autonomic nervous system and neuroendocrine system to regulate inflammation and immunity; *α*7nAChRs are widely expressed in immune cells, neurons, and muscle cells; the activation of *α*7nAChRs can suppress local and systemic inflammation; the expression of *α*7nAChRs represents the acute or chronic inflammatory state to a certain extent; M1AChRs are mainly expressed in the advanced centers of the brain and regulate systemic inflammation; neuroinflammation of the MVZ, hypothalamus, and forebrain induced by sepsis not only leads to their dysfunctions but also underlies the regulatory dysfunction on systemic inflammation and immunity. Correcting the neuroinflammation of these regulatory centers and adjusting the function of *α*7nAChRs and M1AChRs may be two key strategies for the treatment of sepsis in the future.

## 1. Introduction

Critical sepsis still has a very high incidence and mortality [1]. Studies have shown that the inflammatory storm induced by the hyperactivity of innate immunity in the early stage of sepsis [2], the chronic inflammatory state and disorders, or the suppression of innate immunity in the late stage of sepsis [3, 4] underlies the poor survival rate and poor quality of life in septic patients; therefore, correcting the disorders of inflammation and immunity should be a key strategy for the treatment of sepsis.

**Figure 1:**
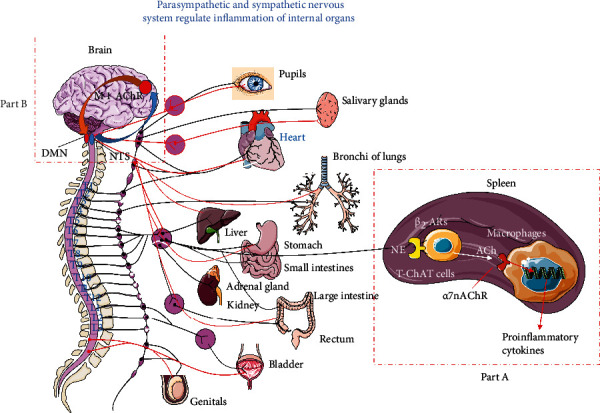
Parasympathetic and sympathetic nervous systems regulate inflammation of internal organs. Sympathetic and parasympathetic systems innervate almost all internal organs. Current studies have confirmed that the vagus nerve can sense inflammatory signals and transmit them to the Nucleus Tractus Solitarius (NTS) of the Medullary Visceral Zone (MVZ). In MVZ, NTS links to the Rostral Ventrolateral Medulla (RVLM) and Vagus Dorsal Motor Nucleus (VDMN) to process the incoming information. On the other hand, MVZ connects the hypothalamus, basal forebrain (BF), and forebrain upwards and connects the locus coeruleus downwards so that the sympathetic and vagus systems, the autonomic nervous system, and the neuroendocrine regulation system can cooperate to regulate inflammation and immunity. (a) The regulation of Cholinergic Anti-inflammatory Pathways (CAP) on systemic inflammation. After the efferent vagus fibers from the Vagus Dorsal Motor Nucleus (VDMN) shift neurons in the upper abdominal cavity mesenteric ganglion complex, their postganglionic fibers enter the spleen and release norepinephrine which acts on the T lymphocytes to facilitate synthesizing ACh, and the latter activate *α*7nAChRs on the monocytes to inhibit releasing proinflammatory cytokines. (b) The central regulation pathway of the cholinergic system. After the inflammatory information from NTS is modified by BF, the postganglionic fibers of BF release ACh which bind to the M1AChRs on the cholinergic neuron of the forebrain to regulate the systemic inflammation and immunity through MVZ.

The autonomic nervous system senses and regulates the functional state including the inflammatory state of internal organs (Figure 1). It innervates, regulates, and activates neutrophils, lymphocytes, and other immune cells to meet the needs of the body's immunity [5]. Overall, the nervous system regulates inflammation and immunity through two main mechanisms [6, 7]: firstly, the neuroendocrine system regulates the release of glucocorticoids through the hypothalamic-pituitary-adrenal (HPA) axis to suppress immunity [8]; and secondly, the Cholinergic Anti-inflammatory Pathway (CAP), belonging to the vagus system, is a rapid and specific regulatory way between the brain and the immune system. CAP includes three parts: the afferent vagus nerve is responsible for perception and incoming inflammatory signals, the Medullary Visceral Zone (MVZ) is in charge of the integration and preliminary treatment of inflammatory signals, and the efferent vagus nerve regulates the strength of inflammation. CAP spontaneously and dynamically regulates the inflammatory tone to ensure effectively preventing tissue damage and infection [9–11]. In addition, the CAP and HPA axis collaborated with each other to maintain a moderate inflammatory and immune state according to the body's needs.

The cholinergic system effectively regulates inflammation and immunity by releasing acetylcholine to act on *α*7nAChRs and M1AChRs (Figure 1). Research confirmed that either M1AChR agonist TBPB or *α*7nAChR agonist GTS-21 can significantly reduce blood proinflammatory cytokine levels such as tumor necrosis factor- (TNF-) *α*, IL-1*β*, and IL-6 in experimental septic mice and reduce mortality [12]. Then, it is necessary to know the differences between these two receptors in the inflammation regulation process. At the same time, sepsis also induces neuroinflammation, which will inevitably affect the structure and function of regulatory centers such as MVZ, and these centers may lose their controlling abilities for inflammation regulation and may be blamed for the inflammatory storm in the early stage of sepsis and the immune paralysis in the late stage. This review collects and analyzes related studies on the cholinergic regulation of inflammation and immunity to generalize the regulatory laws and provide ideas for further research and clinical translation on sepsis.

## 2. Primary Regulation: *α*7nAChRs Mediated the Inflammatory Reflex

Primary regulation involves the direct regulation of systemic and local inflammation by CAP through releasing acetylcholine, which acts on *α*7nAChRs on immune cells or other related cells. Therefore, *α*7nAChRs play key roles in the control of central and peripheral inflammation and are considered key components of the innate immune system [13, 14].

### 2.1. CAP Negatively Regulates Systemic or Local Inflammation and Immunity by means of Activating *α*7nAChRs

There are bidirectional communications between the central nervous system (CNS) and the immune system. Implantable electric vagus nerve stimulation can effectively reduce the blood inflammatory mediators in sepsis [15], which reveals that the vagus is involved in the regulation of inflammation. Further studies have confirmed that CAP participates in inflammation regulation [7, 16]. MVZ, which exists in the midcaudal segment of the medulla oblongata of humans, rats, monkeys, and other animals, is an important regulatory center for stress and an integrating center between the autonomic nervous system and the immune system [17]. MVZ and the vagus nerve constitute a complete circuit for inflammation perception and regulation, which is the so-called CAP. CAP is responsible for monitoring and suppressing systemic or local inflammation and immunity to acquire a balance between removing pathogens and restoring immune homeostasis. The following paths are involved in the regulatory process. The sensory vagus nerve endings sense the inflammatory signals through the aortic body or other chemoreceptors, and the inflammatory signals were transmitted into MVZ or advanced centers such as the forebrain [16]. After the inflammatory signals were integrated and processed among several centers such as the MVZ, hypothalamus, basal forebrain (BF), and forebrain, the final instructions were emanated from the Vagus Dorsal Motor Nucleus (VDMN) and terminated in the upper abdominal cavity mesenteric ganglion complex, from which the adrenergic postganglionic fibers were elongated to the spleen and activate the choline acetyltransferase of T lymphocytes by releasing norepinephrine, which catalyzes the biosynthesis of acetylcholine [13, 18]. Acetylcholine is released and acts on *α*7nAChRs on innate immune cells (such as monocytes) to prevent the activation of monocytes (Figure 1(a)); at the same time, activation of *α*7nAChRs inhibits releasing proinflammatory cytokines from the innate immune cells through the negative regulation of TLR4 signals [19, 20]; thereby, the immunity and inflammation were suppressed in sepsis [21, 22]. In addition to immune regulation through the spleen, the vagus nerve also directly releases ACh and acts on *α*7nAChRs on the intestinal macrophages [23], Kupffer cells in the liver [24], and lung macrophages [25] to curb local inflammation. These studies suggest that the spleen-mediated regulatory path of CAP mainly regulates acute and chronic systemic inflammation such as sepsis, rheumatoid arthritis, and acute pancreatitis, while the vagus system also directly regulates the local inflammation without spleen participation.

### 2.2. The Crosstalk Inside and Outside of MVZ

MVZ is mainly composed of the Nucleus Tractus Solitarius (NTS), Rostral Ventrolateral Medulla (RVLM), Vagus Dorsal Motor Nucleus (VDMN), and other functional nuclei. They are closely related in function and form the primary regulation center of inflammation and immunity. Most of the afferent vagus nerve terminates in NTS and conducts peripheral information to NTS, which was processed by MVZ or transmitted to the advanced center such as BF. NTS, one of the critical centers of the visceral reflex, inhibits the sympathetic activity of RVLM; therefore, the damage of NTS will lead to strong sympathetic output and intensify the inflammation [26]. RVLM is rich in catecholamine neurons, which are roughly divided into three groups. The catecholamine neurons which project to the hypothalamus regulate the release of vasopressin and ACTH, so they can regulate inflammation through the hypothalamic-pituitary-adrenal (HPA) axis; the bulbospinal catecholamine neurons are presympathetic, which regulates most of the visceral functions; the third group of catecholamine neurons regulates parasympathetic efferents to regulate inflammation and immunity [27]. It can be seen that RVLM bridges between the sympathetic and parasympathetic systems, the autonomic nervous and neuroendocrine systems, and the afferent and efferent systems of the vagus to coordinate the regulation of systemic inflammation. VDMN is the ultimate executor of CAP. By optogenetics and functional mapping, it reveals that cholinergic neurons in VDMN project to the superior mesenteric ganglion of the abdominal cavity. The activation of cholinergic neurons in VDMN significantly increases the activity of the splenic nerve and inhibits the production of TNF-*α*, which were canceled by the pharmacology blocking or surgical transection of the vagus nerve, indicating that VDMN negatively regulates innate immunity [28]. These studies confirmed that MVZ is the primary center for regulating systemic inflammation and immunity, especially innate immunity.

MVZ is a transferring station in the communication between the immune system and the brain [29]. NTS and RVLM connect with locus coeruleus (LC), which controls spinal cord-derived catecholaminergic output to participate in immunomodulatory and anti-inflammatory activity; NTS also links to the paraventricular nucleus (PVN) of the hypothalamus; thereby, it regulates inflammation and immunity by affecting the release of glucocorticoids through the HPA axis [30, 31]. Catecholaminergic neurons in MVZ also transmit inflammatory information to the central amygdala nucleus, which regulates the emotional response [32]. In addition, MVZ neurons also project to the BF, and the latter further project to the forebrain and influence the M1 cholinergic neurons in the forebrain; at last, these cholinergic neurons have a significant impact on VDMN [33]. Thereby, the MVZ, BF, and forebrain form a regulatory loop and affect systemic inflammation and immunity through *α*7nAChRs and M1AChRs.

It can be seen that MVZ is the central hub and primary center of the inflammatory reflex. It bridges between the vagus and sympathetic nerves and the autonomic nervous and neuroendocrine systems. It coordinates multiple centers to jointly regulate systemic inflammation and immunity.

### 2.3. The Intracellular Signaling Pathways That Activated *α*7nAChRs Inhibit Inflammation

**Figure 2:**
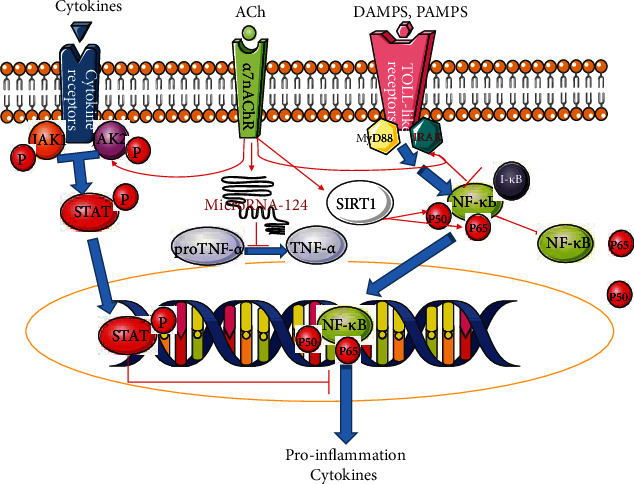
Some known paths of activated *α*7nAChRs to suppress inflammation. Activated *α*7nAChRs inhibit the generation of proinflammatory cytokines through several intracellular paths. These mechanisms involve negatively regulating the NF-*κ*B path and facilitating the synthesis of P-STAT, SIRT1, and microRNA-124. DAMPs: damage-associated molecular patterns; PAMPs: pathogen-associated molecular patterns; JAK2: tyrosine kinase 2; STAT: signal transducer and activator of transcription; IRAK: interleukin-1 receptor-associated kinase. Blue arrow, the normal signal path; red arrow, promoting; red line, blocking.

Activating *α*7nAChRs on macrophages and macrophage cell lines can inhibit them from secreting proinflammatory factors such as IL-1*β*, TNF-*α*, IL-6, and HMGB1 in a concentration-dependent manner but does not affect the secretion of anti-inflammatory cytokines such as IL-10 [34, 35]. The mechanism involves the regulation of multiple signaling pathways. Firstly, activated *α*7nAChRs upregulate the expression of IRAK-M [36], a negative regulator of proinflammatory cytokines, which can inhibit the phosphorylation of upstream signaling molecules of I*κ*B, thereby inhibiting the activity of NF-*κ*B [37]; secondly, through promoting recruitment of tyrosine kinase JAK2 and phosphorylation of STAT3, activated *α*7nAChRs promote pSTAT3 translocation to the nucleus and binding to DNA instead of NF-*κ*B, thereby inhibiting the production of TNF-*α* and other cytokines [38, 39]; thirdly, sirtuin (SIRT) plays an important role in curbing inflammation, and central activated *α*7nAChRs can increase the expression of SIRT1 and deacetylate RelA/p65, thereby promoting RelA/p65 proteasome degradation and reducing cytokine expression [40, 41]; furthermore, activated *α*7nAChRs can upregulate miRNA-124, reduce the expression of IL-6 mRNA, and inhibit the conversion of proTNF-*α* to TNF-*α* to reduce the production of inflammatory factors [42]. *α*7nAChRs are involved in facilitating naive CD4+ T cells to differentiate to CD4+ CD25+ FoxP3+ regulatory T cells (Tregs) [43], and the latter are immunosuppressive T cells and negatively regulate systemic inflammation. From these studies, it can be seen that the activation of *α*7nAChRs can cut off the production of proinflammatory factors through multiple intracellular signaling pathways (Figure 2).

Other studies suggest that *α*7nAChRs have the characteristics of ligand-gated ion channels [44]. In macrophages, PNU-120596, the *α*7nAChRs' allosteric modulator, can increase the opening duration and frequency of the calcium channels, while antagonists reduce these effects of calcium channels induced by choline [45]. As we all know, the content of intracellular calcium will affect a wide range of inflammatory signal paths in the cell; the specific mechanisms that activated *α*7nAChRs regulate inflammation through calcium channels need to be further studied.

### 2.4. The Peripheral Effect of *α*7nAChRs Outside CNS

In sepsis, macrophages and monocytes are first recruited to the site of infection and initiate innate immunity [46]; however, the excessive activation of these cells will cause the inflammatory storm in sepsis [47]. *α*7nAChRs are expressed on the membrane of macrophages, monocytes, and dendritic cells. Activation of *α*7nAChRs can promote the transformation of macrophages from proinflammatory type M1 to anti-inflammatory type M2 [48], which will limit the intensity and extension of inflammation [49]. In addition, to express *α*7nAChRs, neutrophils and T and B lymphocytes also express other nicotinic and muscarinic receptors, which suggests that the vagus regulates immunity through multiple paths in these cells [50]. It can be seen that the excitement of the vagus will influence both innate immunity and acquired immunity, which contribute to the inhibition of systemic inflammation in sepsis.

Besides immune cells, *α*7nAChRs were also expressed on the membranes of peripheral nerve fibers, skeletal muscle, and myocardial cells, which play an important role in controlling the intensity of local inflammation. Sepsis induces a significant upregulation of *γ*- or *α*7nAChRs in the sciatic nerve and causes demyelination and neuromuscular dysfunction [51]; however, the glial cell line-derived neurotrophic factor (GDNF) significantly reduces nerve demyelination, improves neuromuscular dysfunction and patients' prognosis, and reduces the expression of *γ*- or *α*7nAChRs [52]. It suggests that inflammation will facilitate the expression of *α*7nAChRs on the nerve fibers and anti-inflammation will subside their expression. However, other studies suggested that the upregulation of *γ* or *α*7nAChRs on the muscle cell membrane induced by sepsis is the molecular basis of muscle weakness [53]. In the spleen, mesenchymal stem cells downregulate the expression of *α*7nAChRs and reduce the ratio of phosphorylated STAT3 to total STAT3, thereby reducing the intensity of systemic inflammation and preventing organ damage caused by sepsis, as well as in the heart [54]. These studies suggest that sepsis facilitates the expression of *α*7nAChRs in peripheral tissue cells, which is related to local or systemic inflammation regulation. In fact, local pathological processes such as cell degeneration and apoptosis should result from systemic or local inflammation rather than upregulated *α*7nAChRs. Upregulated *α*7nAChRs may be a marker for local inflammation and pathologies of degeneration and apoptosis.

### 2.5. The Central Effect of *α*7nAChRs

*α*7nAChRs are encoded by the gene fragment of CHRFAM7A. They were first found on neurons and were most richly expressed in CNS and involved in the regulation of neuroinflammation. Intracerebral injection of the *α*7nAChR agonist PHA-543613 reduces neuroinflammation by activating the JAK2-STAT3 pathway, thereby lessening the short-term and long-term sequelae after intracranial hemorrhage (ICH), while the *α*7nAChR antagonist MLA has the opposite effect [55]; Transcutaneous vagus nerve stimulation (VNS) also has the effect of lessening brain damage after stroke which may be related to the upregulation of *α*7nAChRs on the membrane of immune cells and neurons [56]. Activated *α*7nAChRs on microglia and macrophages, which infiltrate the brain through the damaged blood-brain barrier, can reduce not only the local inflammatory cytokines such as TNF-*α* and IL-1*β* but also the death of the neurons in the hippocampal CA1 zone [57]. PHA568478, the selective *α*7nAChR agonist, can reduce the number of microglia/macrophages of the proinflammatory M1 phenotype and increase the number of microglia/macrophages of the anti-inflammatory M2 phenotype [58]. It shows that *α*7nAChR activation of central immune cells or neurons, either by the direct agonist or by the indirect peripheral VNS, can produce central anti-inflammatory effects [59]. In addition to regulating acute or chronic neuroinflammation, activation of *α*7nAChRs is involved in improving cognitive function, mental health, and neurodegenerative diseases, even energy homeostasis and insulin sensitivity [60], and interfering with *α*7nAChRs has broad prospects for clinical application.

Our preliminary experiments confirmed that activation of *α*7nAChRs significantly improved MVZ neuroinflammation and apoptosis [61, 62]: in sepsis, intraperitoneally administered GTS-21, a specific agonist for *α*7nAChRs, significantly reduced the apoptosis of the cholinergic and catecholaminergic neurons in MVZ in septic rats, accompanied by the elevated expression of tyrosine hydroxylase (TH) and choline acetyltransferase (CHAT), which significantly reversed the inhibition of CAP and the inflammatory storm, while intraperitoneally administered MLA, a specific antagonist for *α*7nAChRs, does the opposite. Therefore, activation of *α*7nAChRs not only directly inhibits the overactive systemic inflammation and immunity but also resumes the inflammatory and dysfunctional MVZ, thereby restoring CAP's regulation of inflammation. Our studies show that through the central and peripheral mechanisms, activation of *α*7nAChRs coordinately promotes the abnormal inflammatory state of sepsis to return to normal.

In addition, research suggests that the centrally administered *α*7nAChR agonist can significantly reduce the multiple organ damage caused by sepsis, reverse the immunosuppressive state, improve the outcome of septic rats [63], reduce intestinal inflammation and the incidence of postoperative intestinal obstruction [64, 65], inhibit the proinflammatory phenotype of microglia activated by traumatic brain injury (TBI), and attenuate the systemic inflammatory response [66], while centrally administered methyllycaconitine, a selective antagonist of *α*7nAChRs, can exacerbate systemic inflammation and multiple organ dysfunction.

These studies have verified that CAP activation by peripheral or central paths can improve the pathology of inflammation regulation centers such as MVZ and restore their normal regulation activity in sepsis, which means central anti-inflammation may be an important strategy to reverse the inflammatory storm in the early stage of sepsis.

## 3. Advanced Regulatory Mechanism: Modification and Regulation of Inflammatory Information by Activating M1AChRs

### 3.1. Basal Forebrain “Modifies” the Afferent Signal of MVZ and Regulates the MVZ Function through the Forebrain

The basal forebrain (BF) acts as a “goalkeeper” or a “filter” in the process of a variety of incoming sensory information [67]. In BF, cholinergic neurons account for only 5% of the total neurons, while GABAergic neurons account for 35%, and glutamatergic neurons account for 55% [68]. Such a neuronal composition allows BF to regulate the afferent intensity of various sensory information easily [69]; namely, the BF cholinergic neurons will amplify or weaken part of incoming sensory information and plastically process them according to the body's needs or the “importance” of these signals [70]. A study shows that inflammatory information transmitted to NTS via the vagus nerve succeeds to BF. After being modified by BF, the inflammatory information were carried by the cholinergic efferent fibers of BF and projected to the M1AChRs richly expressed in the forebrain [71]. The activation of M1AChRs in the forebrain will inhibit systemic inflammation and immunity [4, 72]. These studies suggest that BF, as the advanced processing center of inflammatory information, actively participates in the inflammatory reflex of MVZ through the forebrain M1AChR system.

### 3.2. Activation of M1AChRs in the Forebrain System Regulates Inflammation and Immunity of Sepsis through CAP

M1AChRs are G protein-coupled receptors with high selectivity [32]. A study [73] confirmed that intraperitoneal injection of cholinesterase inhibitors such as galantamine or Huperzine A, which can pass through the blood-brain barrier, significantly reduced plasma proinflammatory cytokines and mortality in septic rats, whereas intraperitoneal injection of cholinesterase inhibitors which cannot pass through the blood-brain barrier has no such effect. This study shows that central acetylcholine has the effect of inhibiting systemic inflammation. A study also shows that intraperitoneal administration of benzyl quinolone carboxylic acid (BQCA, positive allosteric regulator of M1AChRs, which can increase the affinity of ACh and M1AChRs by 129 times in CNS [74]) can reduce the serum TNF-*α* levels, and it indicates that through activation of the central M1AChRs, central acetylcholine has a negative regulation on inflammation in sepsis, which may be related to the inflammation-controlling loop of BF-forebrain-MVZ.

In addition, direct activation of central M1AChRs also has a significant anti-inflammatory effect. For example, administration of M1AChRs' specific agonist CNI-1493 in the brain inhibits serum TNF-*α* in murine endotoxemia [75]. M1AChRs are richly expressed in the forebrain. Should the forebrain M1AChRs be responsible for this anti-inflammatory function? The next research gave us the answer. In the forebrain, selective genetic ablation of the vesicular acetylcholine transporter (VAChT, which is necessary for the release of acetylcholine) (VAChT-/- mice) eliminates the synaptic release of acetylcholine (ACh) and leads to increased serum TNF-*α* levels [76]. From these studies, we can affirm that activating the forebrain M1AChRs is a key step to control systemic inflammation. Should the anti-inflammatory effect of the forebrain M1AChRs be linked to CAP? The following studies revealed the answer. Intracerebroventricular injection of McN-A-343, the M1AChR agonist, reduced the severity of ulcerative colitis, but the effect was eliminated by splenectomy [77]; other studies also confirmed that the anti-inflammatory effect by the activation of M1AChRs can be abolished by CAP disconnection [78, 79]. These studies suggest that activating forebrain M1AChRs can inhibit systemic inflammation by means of CAP. A study also confirmed that the forebrain has bidirectional fiber connections with NTS [80]. Therefore, it can be certain that the regulatory function of M1AChRs in the forebrain on inflammation and immunity is through MVZ and CAP [81] (Figure 1(b)).

## 4. Neuroinflammation Induced by Sepsis Causes Extensive Damage to the CAP's Regulation on Inflammation and Immunity

LPS can activate microglia and astrocytes to promote the expression of various proinflammatory cytokines and lead to CNS inflammation. Proinflammatory cytokines affect the function of neurons and boost neuronal apoptosis, which will cause neuroregulation disorders [82, 83]. In sepsis, neither BF nor MVZ cannot escape from the attack of sepsis-induced neuroinflammation [84, 85]. In MVZ, neuroinflammation has a comprehensive influence on nerves architecture, synaptic plasticity, and nerve function. Studies show that cholinergic and catecholaminergic neurons in MVZ went through significant apoptosis and inactiveness, which contributes to the inhibition of CAP and the acceleration of the inflammatory storm in early sepsis [61, 62]. Neuroinflammation facilitates reactive glial cells in the brain, releasing cytokines and damage-associated molecular patterns (DAMPs), which promote cell adhesion, migration, proliferation, and angiogenesis [86]. In sepsis, activation of the HMGB1-RAGE axis upregulates TH expression in dopaminergic neurons [87, 88] and aggravates oxidative stress, Ca^2+^ homeostasis disruption, endoplasmic reticulum stress, autophagic dysfunction, excitotoxicity, and free-radical generation, all of which could lead to apoptosis [89, 90]. Both inflammation and oxidative stress in the hypothalamus can lead to excessive activation of paraventricular nucleus (PVN) neurons which project to RVLM, thereby triggering sympathetic excitation and blood pressure elevation [91, 92]. Furthermore, the attack by LPS in the hypothalamus can lead to neuroinflammatory and metabolic disorders, which lead to the downregulation of *α*7nAChRs and the decreased activation of STAT3 and worsen neuroinflammation [93, 94].

In addition, inflammation upsets the balance of neurotransmitters between monoamine and glutamate, which leads to decreased functional connectivity among multiple brain regions around the ventromedial prefrontal cortex (vmPFC) [95]. Inflammation also leads to synaptic dysfunction. IL-10, one of the main anti-inflammatory cytokines, can recover multiple neurofunction in sepsis [96]. The stress caused by sepsis increases sympathetic tension and decreases parasympathetic tension, thereby further aggravating neuroinflammation and affecting the functions of the prefrontal cortex, hippocampus, amygdala, and septal areas [97]. It can be seen that the neuroinflammation of MVZ, forebrain, and hypothalamus in sepsis will inevitably affect their inflammation regulation function, which may be a more important mechanism of systemic inflammation and immune disorders induced by sepsis.

## 5. Regulation of the Sympathetic System on Inflammation and Immunity

As mentioned above, MVZ communicates upward with the hypothalamus, the sympathetic nerve center, and communicates downward with the locus coeruleus (LC) to further affect the sympathetic neurons of the spinal cord, both of which are involved in the regulation of systemic inflammation. Besides participating in CAP to regulate inflammation [18, 98], the sympathetic system can also independently regulate innate immunity and adaptive immunity [99]. Sympathetic nerves act on invariant NKT cells in the liver and turn them from the proinflammatory Th1 type into the anti-inflammatory Th2 type to promote immunosuppression by releasing IL-10, which can easily lead to bacterial infections [100, 101]. Sympathetic nerves affect the innate and adaptive immunity by stimulating adrenergic receptors; the activation of *β*2-adrenergic receptors has immunosuppressive effects on monocytes and macrophages [102, 103]. However, NE and *β*2 agonists activate *β*2-adrenergic receptors on lymphocyte B cells to increase antibody production to enhance adaptive immunity [104]. Existing research studies are more inclined to support that the sympathetic system participates in stress and promotes inflammation, whereas vagus nerves inhibit inflammation and prevent tissue damage.

## 6. Conclusion

The autonomic nervous system, especially the vagus system, plays a key role in regulating inflammation and immunity in sepsis through *α*7nAChRs and M1AChRs. *α*7nAChRs are expressed on central and peripheral cells to regulate local or systemic inflammation; M1AChRs are expressed mainly in the advanced center and regulate systemic inflammation and immunity through MVZ. MVZ is the CAP's center, which communicates between the sympathetic and vagus systems, the autonomic nervous system, and the neuroendocrine system to regulate inflammation and immunity; the neuroinflammation of the regulation center may be a more noteworthy mechanism why inflammation and immune disorders are difficult to correct in sepsis.
